# Biomechanical effects of different numbers and locations of screw-in clavicle hook plates

**DOI:** 10.3389/fbioe.2022.949802

**Published:** 2022-09-09

**Authors:** Cheng-Chi Wang, Cheng-Hung Lee, Kun-Hui Chen, Chien-Chou Pan, Ming-Tzu Tsai, Kuo-Chih Su

**Affiliations:** ^1^ Department of Orthopedics, Taichung Veterans General Hospital, Taichung, Taiwan; ^2^ Department of Health Services Administration, China Medical University, Taichung, Taiwan; ^3^ Department of Public Health, China Medical University, Taichung, Taiwan; ^4^ Department of Food Science and Technology, HungKuang University, Taichung, Taiwan; ^5^ Department of Post-Baccalaureate Medicine, College of Medicine, National Chung Hsing University, Taichung, Taiwan; ^6^ Department of Computer Science and Information Engineering, Providence University, Taichung, Taiwan; ^7^ Department of Rehabilitation Science, Jenteh Junior College of Medicine, Nursing and Management, Miaoli, Taiwan; ^8^ Department of Biomedical Engineering, HungKuang University, Taichung, Taiwan; ^9^ Department of Medical Research, Taichung Veterans General Hospital, Taichung, Taiwan; ^10^ Department of Chemical and Materials Engineering, Tunghai University, Taichung, Taiwan

**Keywords:** clavicle hook plate, biomechanics, finite element analysis, acromioclavicular joint, numbers of screw, locations of screw

## Abstract

**Purpose:** We sought to analyze the biomechanical effects which both different numbers and locations of screws have on three different clavicle hook plates, as well as any possible causes of sub-acromial bone erosion and peri-implant clavicular fractures.

**Methods:** This study built thirteen groups of finite element models using three different clavicle hook plates (short plates, long plates, and posterior hook offset plates) in varying numbers and locations of the screws. The von Mises stress distribution of the clavicle and hook plate, as well as the reaction force of the acromion was evaluated.

**Results:** The results show that inserting screws in all available screw holes on the hook plate produces a relatively large reaction force on the acromion, particularly in the axial direction of the bone plate. The fewer the screws implanted into the clavicle hook plate, the larger the area of high-stress distribution there is in the middle of the clavicle, and also, the higher the stress distribution on the clavicle hook plate.

**Conclusion:** This study provides orthopedic physicians with the biomechanical analysis of different numbers and locations of screws in clavicle hook plates to help minimize surgical complications.

## Introduction

Clavicle hook plates are commonly used for acromioclavicular joint dislocations and distal clavicle fractures (Di Francesco et al., 2012; Tiren et al., 2012; Kumar and Sharma, 2015; Yoon et al., 2018; Baunach et al., 2021). During surgery, after the reduction of a fracture or dislocation, the hook is applied under the acromion and the plate is fixed by screws on the clavicle. Hook plates are available in various lengths and offsets to suit different anatomies and clinical conditions. Hook plates provide a convenient and reliable method, as well as good stability for the fixation of the acromioclavicular joint (Kim et al., 2015).

Stress on both the acromion and clavicle changes after implantation of a hook plate. Common complications resulting from hook plates include sub-acromial bone erosion (Chiang et al., 2010; Hoffler and Karas, 2010; Lopiz et al., 2019) and peri-implant clavicular fractures (Charity et al., 2006; Ding et al., 2011; Lopiz et al., 2019; Ni et al., 2020; Shih et al., 2020), which are related to changes in stress on the bone after implantation. Understanding these stress changes is essential for physicians. However, there is currently no consensus on the optimal number of screws or screw locations on the plate. Sometimes the screw hole is at the fracture site, or the screw position is eccentric due to the curvature of the plate not matching the curvature of the clavicle. Therefore, not all screw holes are necessarily secured with screws. There have not yet been any clinical studies or biomechanical experiments to investigate the effects of different screw numbers and their locations.

Finite element analysis (FEA) is often used in orthopedic studies in order to analyze the biomechanical effects of different material properties and the different geometric shapes of plates (Marinescu et al., 2017; Hamandi et al., 2018; Antoniac et al., 2019). According to previous studies, lower-offset hook plates and shorter plates increase subacromial stress (Shih et al., 2015; Lee et al., 2016). A larger hook angle increases subacromial stress but reduces stress around the plate (Hung et al., 2017). Therefore, FEA is suitable for evaluating the impact a hook plate has on the shoulder after implantation.

This study conducted FEA in order to analyze the biomechanical effects of different types of hook plates in varying screw numbers and locations, as well as the possible causes of sub-acromial bone erosion and peri-implant clavicular fractures. Orthopedic physicians will be able to utilize the study results to better place screws in their appropriate positions to help reduce surgical complications.

## Materials and methods

### Building a simulation geometry model

**FIGURE 1:**
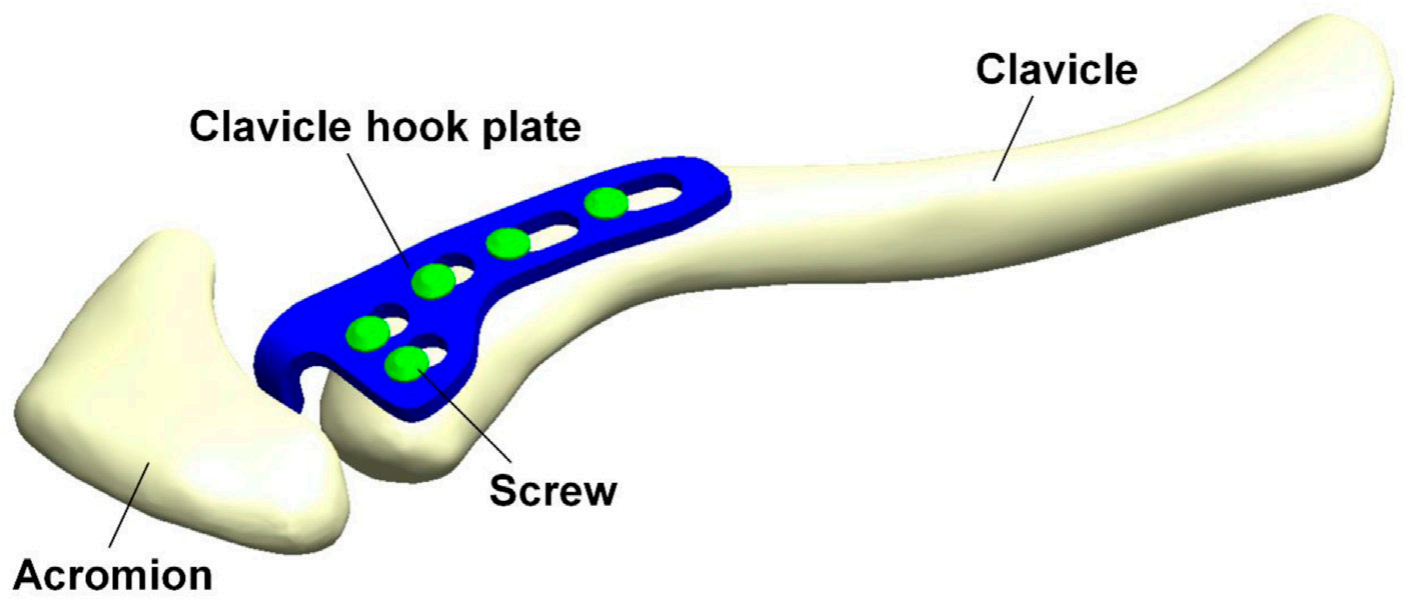
Computer model for finite element analysis of a clavicle hook plate implanted in an acromioclavicular joint.

This study involved building a computer model for FEA of clavicle hook plates which had been implanted in an acromioclavicular joint (Figure 1) for the purposes of investigating the effects different screw numbers and locations of different clavicle hook plates have on patients. The model used in this study was divided into four parts: the clavicle, acromion, clavicle hook plate, and screws. The models of the clavicle and acromion used CT images provided by the United States National Library of Medicine’s Visible Human Project, and then used Mimics software (Mimics Medical 20.0, Materialise, Leuven, Belgium) to select the clavicle and acromion. The bone was then divided into two parts: the cortical bone and cancellous bone. In addition, this study used CAD software Solidworks 2016 (Solidworks 2016, Dassault Systemes SolidWorks Corp, Waltham, MA, United States) to build the models of the clavicle hook plates and screws.

**FIGURE 2:**
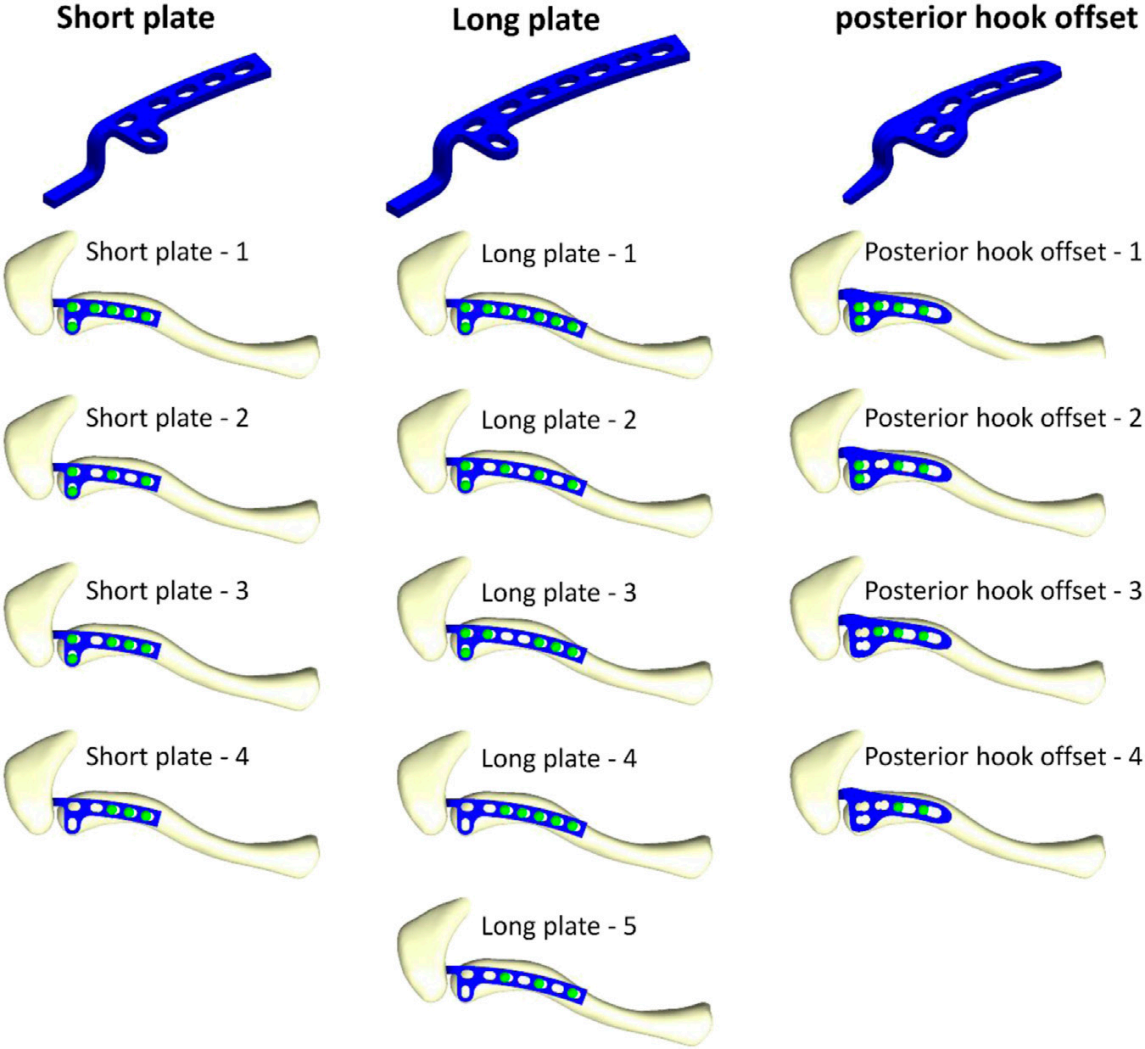
Thirteen models composed of three different clavicle hook plates, with various screw placement positions.

According to the existing clavicle hook plate system used in clinical practice, three different clavicle hook plate models are then established: the short plate, long plate, and posterior hook offset plate (Figure 2). The number of screws that can be implanted in the three groups of clavicle hook plates are six-hole, eight-hole, and five-hole, respectively. Therefore, different numbers of screws were implanted in the three different models of the clavicle hook plates, which could then be further divided into thirteen groups (Figure 2). CAD software Solidworks was utilized to combine the clavicle, acromion, clavicle hook plate, and screws. Therefore, this study built a total of thirteen groups of computer finite element models. After the 3D finite element models were established, the models were imported into FEA software (ANSYS Workbench 18.0, ANSYS, Inc., Canonsburg, PA, United States) for finite element analysis simulation.

### Boundary conditions and load conditions

**FIGURE 3:**
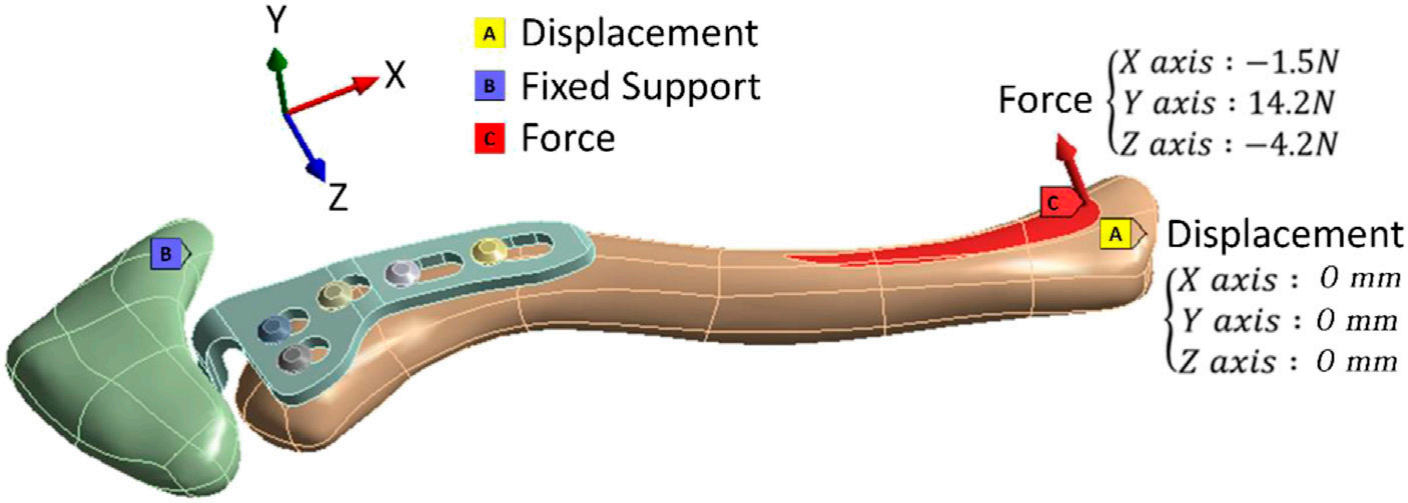
Boundary conditions and load conditions.

The boundary conditions and load conditions in this study were determined by simulating the force of the sternocleidomastoid muscle when the arm picked up a teacup (the static position in front of the mouth with a cup weighing 0.5 kg in the hand) (Cronskär et al., 2015). Therefore, one load condition and two boundary conditions were given. The load condition is determined when a pull-up force is applied to the area where the sternocleidomastoid muscle attaches to the clavicle (X-axis: −1.5°N, Y-axis: 14.2°N, Z-axis: −4.2°N). The boundary condition is that the lower ends of the proximal clavicle and acromion are set to be fixed. The proximal clavicle is fixed at one point (the X-axis, Y-axis, and Z-axis displacements of this point are set to zero) so that the clavicle can rotate after being stressed (Figure 3). In addition, this study mainly simulates the clavicle hook plate implantation when the acromioclavicular ligament and the coracoclavicular ligament have been ruptured, so external force was only applied to the sternocleidomastoid muscle. In this study, the clavicle was built using CAD software. Therefore, each clavicle was placed in the same position in the computer (the clavicle position was also determined by reference to the previous study). As a result, it was possible to provide the loading conditions for each group in this study by referring to the external force from the previous study. In addition, in order to make this study closer to the actual situation, the contact between the clavicle hook plate and screws is set as “bonded,” and the contact between the clavicle hook plate and acromion is set as “no separation.” “No separation” means that two faces in non-separating contact are in contact only in their normal direction, allowing a slight slither between each other in the tangential direction (Lee, 2018). Such a setting can simulate the actual situation of hook plate implantation being performed in the human body.

### Material properties of the model

**FIGURE 4:**
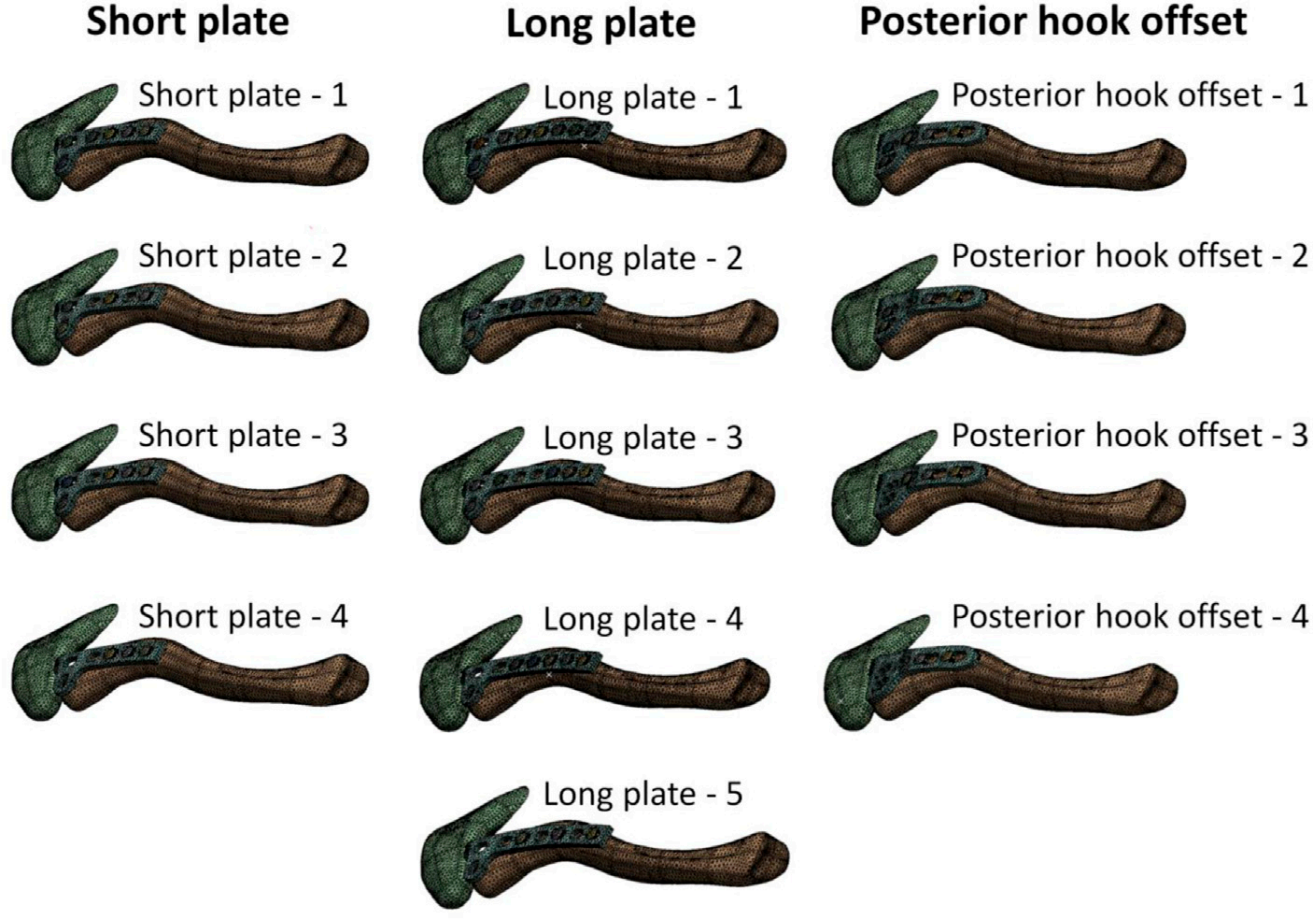
The 13 groups of models after meshing.

The computer model of this study consisted of four parts: the clavicle, acromion, clavicle hook plate, and screws. The material of the clavicle hook plate was simulated in titanium. All materials were assumed to be homogeneous, isotropic, and linearly elastic. According to previous studies, Young’s modulus (E) and Poisson’s ratio (ν) were used to determine the material properties (Table 1) (Shih et al., 2015; Lee et al., 2016; Hung et al., 2017). The mesh element used in the finite element analysis computer model of this study is a tetrahedral mesh. A convergence test was performed prior to finite element analysis in order to make computer simulation data more accurate. The convergence test used in this study was mainly controlled by mesh size as the basis for convergence. The mesh sizes controlled by the convergence test were 3, 2, 1, 0.9, and 0.8 mm, respectively. After the mesh convergence test, the mesh size was 0.9 mm and the model reached 5% of the stopping criterion for the convergence test. The thirteen groups of models were meshed at a mesh size of 0.9 mm and built using quadratic tetrahedral elements (Figure 4). Therefore, it is reasonable to use the mesh model in this study to investigate the biomechanical influence of different numbers and locations of screws in clavicle hook plates. Table 2 shows the number of elements and nodes after meshing in each group.

**TABLE 1:** Material properties of this study.

Material	Young’s modulus (MPa)	Poisson’s ratio
Cortical bone	17,000	0.3
Cancellous bone	1,000	0.3
Clavicle hook plate	200,000	0.3
Screws	118,000	0.3

**TABLE 2:** The number of elements and nodes after meshing in each model.

	Short plate—1	Short plate—2	Short plate—3	Short plate—4	
Nodes	191,553	175,715	183,417	168,152	
Elements	105,715	96,950	101,149	92,739	
	**Long plate—1**	**Long plate—2**	**Long plate—3**	**Long plate—4**	**Long plate—5**
Nodes	612,485	596,956	602,359	595,918	584,471
Elements	404,098	397,039	399,433	396,073	390,504
	**Posterior hook offset—1**	**Posterior hook offset—2**	**Posterior hook offset—3**	**Posterior hook offset—4**	
Nodes	184,721	177,315	168,800	162,032	
Elements	101,939	97,890	93,088	89,453	

After finite element analysis, this study observed von Mises stress in different types of clavicle hook plates involving different screw numbers and locations. The stress distribution on the clavicle hook plate and clavicle, as well as the reaction force of the acromion, was analyzed.

## Results

**FIGURE 5:**
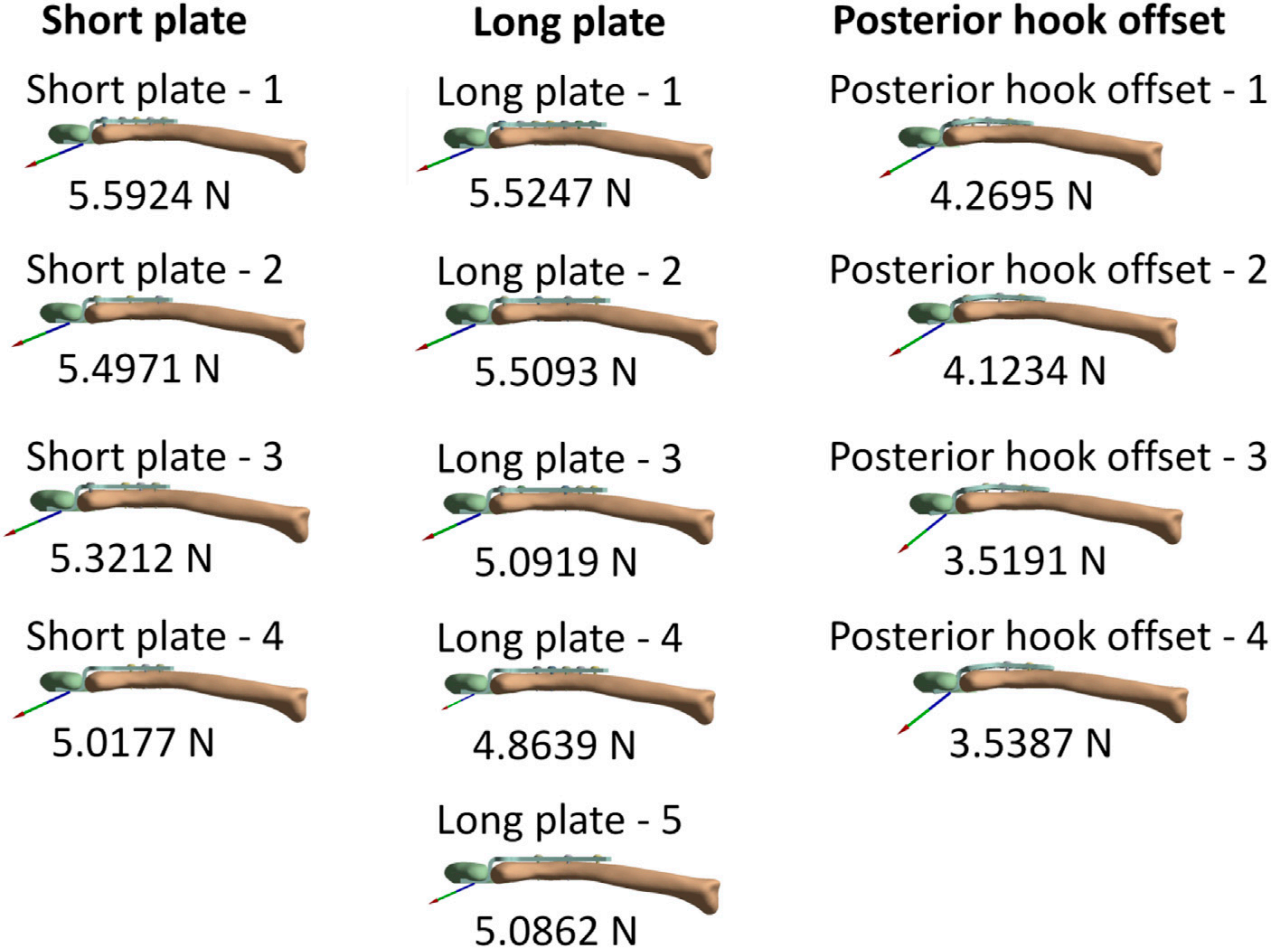
Force reaction on the acromion.

Figure 5 shows the force reaction of the acromion. It reveals that there is a relatively small reaction force on the acromion where the screw holes near the acromion have no screws (short plate—4, long plate—4, long plate—5, posterior hook offset—3, and posterior hook offset—4). Table 3 shows the magnitude of force reaction on the acromion in the X-, Y-, and Z-axes. For the clavicle hook plates having shorter lengths (five-hole and six-hole plates), the X-axis component force increases with the number of implanted screws. The component forces in the Y-axis and the Z-axis do not differ significantly.

**TABLE 3:** Force reaction on the acromion and component forces in the X-, Y-, and Z- axes.

Force reaction (N)	X axis	Y axis	Z axis (N)	Total (N)
Short plate—1	−5.1803°N	−2.1056°N	0.073909	5.5924
Short plate—2	−5.0915°N	−2.0708°N	0.084036	5.4971
Short plate—3	−4.8796°N	−2.119°N	0.11655	5.3212
Short plate—4	−4.5425°N	−2.1171°N	0.24543	5.0177
Long plate—1	−5.1169°N	−2.0816°N	0.080534	5.5247
Long plate—2	−5.0486°N	−2.2029°N	0.10746	5.5093
Long plate—3	−4.6323°N	−2.1068°N	0.17601	5.0919
Long plate—4	−4.3756°N	−2.1037°N	0.29306	4.8639
Long plate—5	−4.6381°N	−2.0717°N	0.25604	5.0862
Posterior hook offset—1	−3.7035°N	−2.1063°N	0.27617	4.2695
Posterior hook offset—2	−3.517°N	−2.1286°N	0.31965	4.1234
Posterior hook offset—3	−2.712°N	−2.1803°N	0.52533	3.5191
Posterior hook offset—4	−2.7474°N	−2.1657°N	0.53283	3.5387

**FIGURE 6:**
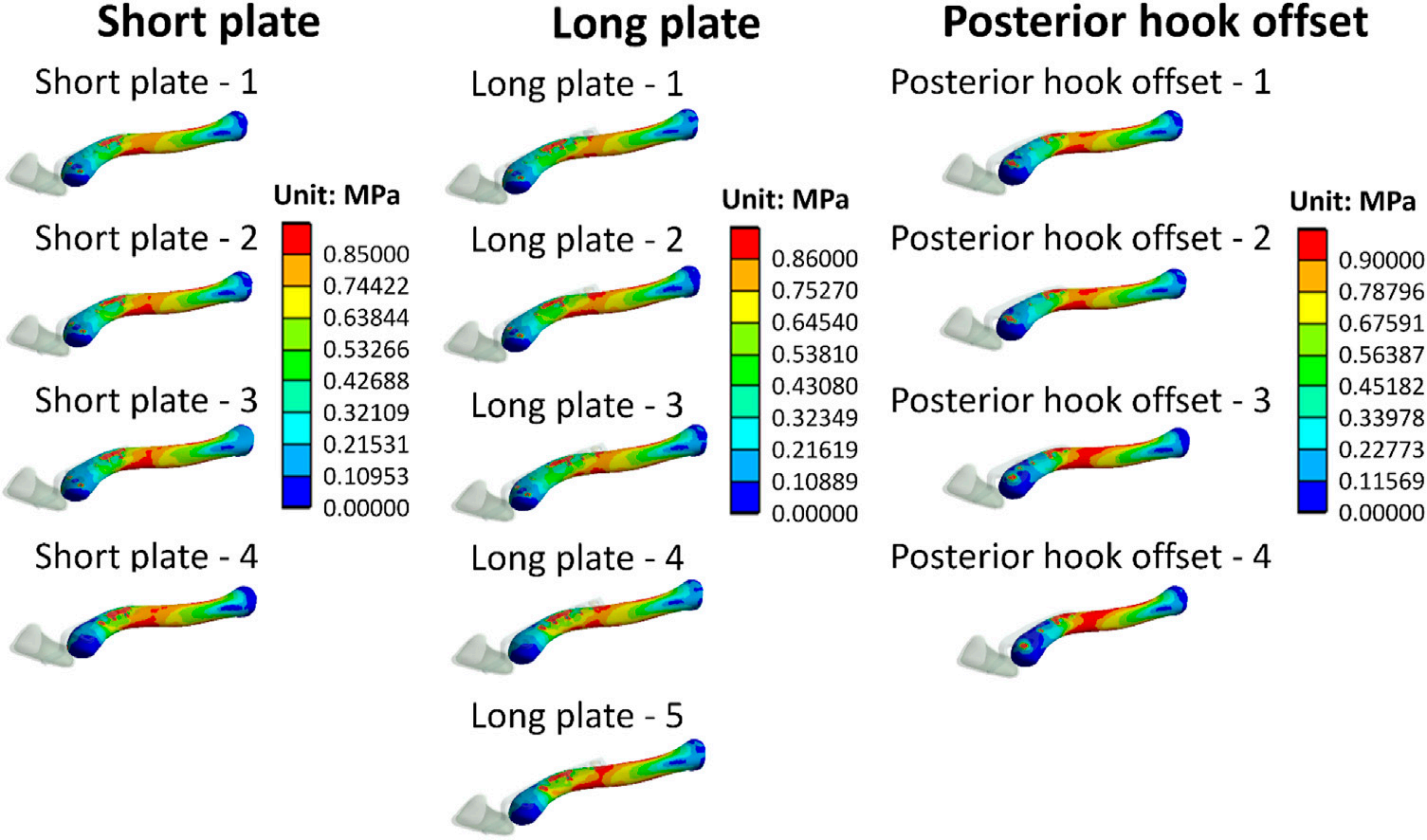
Stress distribution of clavicles.

Figure 6 shows stress distribution on the clavicle. When more screws are implanted on the clavicle hook plate, the smaller the area of high stress on the clavicle.

**FIGURE 7:**
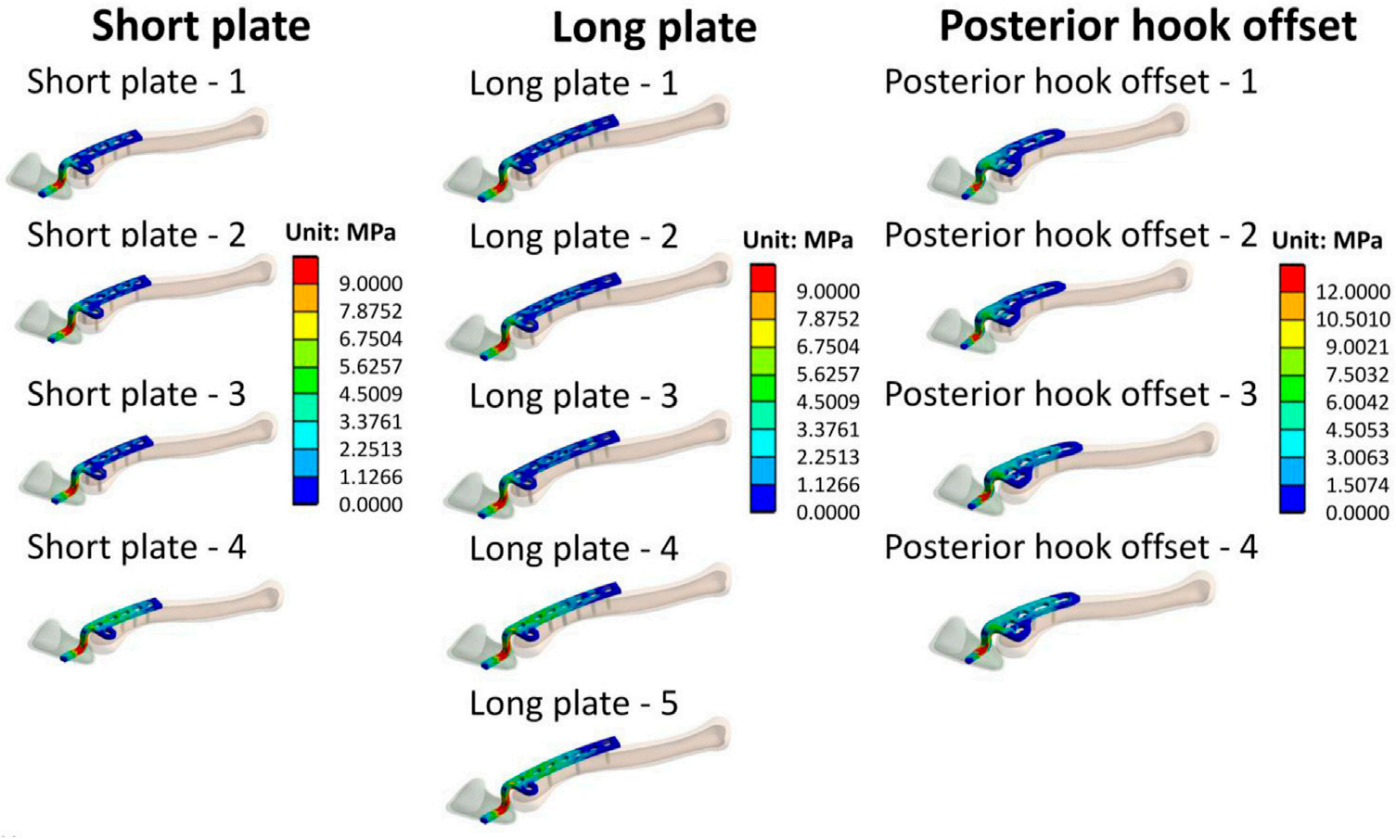
Stress distribution on clavicle hook plates.

Figure 7 shows stress distribution of the clavicle hook plates. It can be seen that when there are fewer screws implanted on the clavicle hook plate, high stress on the clavicle hook plate occurs.

## Discussion

In this study, finite element analysis was used to investigate both the influence and biomechanical effects of different clavicle hook plates with different screw fixation methods. The results of this study can provide valuable biomechanical references for orthopedic physicians.

**FIGURE 8:**
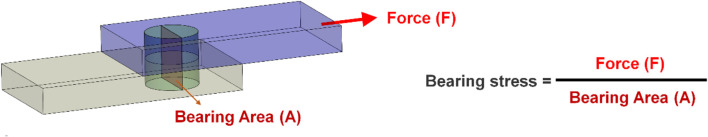
The force of bearing stress.

When all screw holes on the clavicle hook plate have implanted screws (group short plate—1, long plate—1, and posterior hook offset—1), there is a relatively large force reaction on the acromion. However, when observing the force reaction in each group and component forces in the X-, Y-, and Z- axes, it can be seen that the component forces in the Y-axis and Z-axis do not differ significantly. Therefore, the force reaction notably changes in the X-axis. The X-axis is parallel to the long axis of the clavicle hook plates. The greater the number of implanted screws, the more resistant force is to the external force in the X-axis direction. The X-axis component force on the acromion is based on how the bearing stress resists the force. When bearing stress = force/bearing area, then force = bearing stress × bearing area (Figure 8). When more screws are implanted, the bearing area will be larger; therefore, the external force on the X-axis of the acromion is also greater. The increased external force on the acromion increases the risk of sub-acrominal bone erosion.

In addition, von Mises stress distribution on the clavicle was observed. Each group displayed lower stress in the area where the clavicle hook plate was implanted. The main reason for this is that after the plate was implanted, a stress-shielding effect in the area of the plate on the bone occurred. In turn, the force that the bone should bear was shielded by the implant; therefore, the clavicle bone experienced a lower stress distribution in the area covered by a plate. In addition, it was found that when the number of implanted screws was fewer in number, the greater the stress that was generated near the screws. The main reason for this is that the smaller the number of implanted screws, the smaller the contact area between the screws and the clavicle, which in turn results in higher stress. In addition, it was found that high stress was generated in the middle segment of the clavicle in each group. The main reason for this is that the cross-sectional area of the middle clavicle region is relatively small, so when the clavicle is subjected to external force, there will be a higher stress value. In addition, it was revealed that the smaller the number of screws implanted on the clavicle hook plate (group short plate—4, long plate—5, and posterior hook offset—4), the larger the area of high-stress distribution in the middle of the clavicle. The main reason for this is that the number of implanted screws was fewer, making the overall structure unstable, in turn resulting in a large displacement on the clavicle. According to Hooke’s law, stress is proportional to strain. The fewer the number of screws implanted on the clavicle hook plate, the higher the stress will be on the clavicle. The higher stress on the clavicle bone increases the risk of peri-implant clavicular fracture.

Stress distribution on the clavicle hook plate can be observed in Figure 7. It is shown here that each group has higher stress at the angle of the hook. The main reason for this is that it is at the corner of the geometric shape, causing the phenomenon of stress concentration. Such results are the same as those seen in previous studies (Shih et al., 2015; Lee et al., 2016; Hung et al., 2017). In addition, observing the implantation of different numbers of screws in each group, it was revealed that when the number of screws is less, there will be an area with a higher stress distribution on the clavicle hook plate. In addition, according to previous studies (Marinescu et al., 2017), the design of holes in the bone plate may cause the bone plate to break. Therefore, when designing the bone plate, unnecessary holes in the bone plate must be avoided. The main reason for this is that after the bone plate is implanted, a stress-shielding effect on the bone in the area of the bone plate occurs. Fewer the screws that are implanted, the larger the area of the plate that transmits force.

There are many limitations surrounding the finite element analysis performed in this study. The material properties are assumed to be homogeneous, isotropic, and linearly elastic. Clavicle bones are not homogeneous, isotropic, and linearly elastic. However, the focus of this study was to assess the impact of different numbers and locations of the screws in clavicle hook plates. Therefore, only the representative clavicle bone models were selected for analysis, and the bone material setup was simplified in this study. We would also like to use anisotropic material properties or different bone material properties to set up bone tissue (Cicciù et al., 2018). However, the finite element analysis of non-linear material properties is difficult to solve. Therefore, the non-linear material or different bone material properties setup used in this study may show more influencing factors on the topics covered in this study. Therefore, this study established cortical and cancellous bones on the basis of most previous finite element analysis studies (Hamandi et al., 2018). In addition, this study mainly simulates the clavicle hook plate implantation when the acromioclavicular ligament and the coracoclavicular ligament have been ruptured, so external force was only applied to the sternocleidomastoid muscle. This setting was based on previous research studies (Shih et al., 2015; Lee et al., 2016; Hung et al., 2021). In addition, the scapula model was simplified, so only the acromion was shown. The scapula model was simplified to reduce the computer calculation time. In addition, although in terms of contact setting, if there is a good setting, the research is closer to the actual situation (Chirca et al., 2021). In order to avoid increasing the parameters discussed in this study, the contact between the clavicle hook plate and acromion is set as no separation. The contact surface between the bone plate and the screw is bonded. The main purpose was to simulate the effect when the bone plate is the locking plate to avoid the factors of instability in the screw–plate contact.

Although some differences can be seen between the simulation in this study and the actual clinical situation, orthopedic physicians can still use the results to better understand the impact that different clavicle hook plates have in variable screw fixation methods on an acromioclavicular joint, as well as assist in reducing post-implantation surgical failures and complications. The study can also provide a reference for the design of new clavicle hook plates in the future.

## Conclusion

Finite element analysis evaluates the biomechanical effects of different clavicle hook plates and different screw fixation methods. Our results show that inserting screws in all available screw holes on the hook plate produces a relatively large force reaction on the acromion, particularly in the axial direction of the bone plate. The fewer the screws implanted into the clavicle hook plate, the larger the area of the high stress distribution that can be seen in the middle of the clavicle, and also the higher the stress distribution on the clavicle hook plate.

## Data Availability

The raw data supporting the conclusion of this article will be made available by the authors, without undue reservation.
